# Selective Binding of HSC70 and its Co-Chaperones to Structural Hotspots on CFTR

**DOI:** 10.1038/s41598-020-61107-x

**Published:** 2020-03-06

**Authors:** Imad Baaklini, Conrado de Campos Gonçalves, Gergely L. Lukacs, Jason C. Young

**Affiliations:** 1 McGill University, Department of Biochemistry, Montreal, H3G 1Y6 Canada; 2 McGill University, Department of Physiology, Montreal, H3G 1Y6 Canada

**Keywords:** Chloride channels, Ion channels, Protein folding, Chaperones, Peptides, Chaperones, Membrane proteins, Chaperones, Endoplasmic reticulum, Endocytosis, Endoplasmic reticulum, Membrane trafficking, Protein folding

## Abstract

Mutations in the cystic fibrosis transmembrane conductance regulator (CFTR) channel cause cystic fibrosis. Chaperones, including HSC70, DNAJA1 and DNAJA2, play key roles in both the folding and degradation of wild-type and mutant CFTR at multiple cellular locations. DNAJA1 and HSC70 promote the folding of newly synthesized CFTR at the endoplasmic reticulum (ER), but are required for the rapid turnover of misfolded channel at the plasma membrane (PM). DNAJA2 and HSC70 are also involved in the ER-associated degradation (ERAD) of misfolded CFTR, while they assist the refolding of destabilized channel at the PM. These outcomes may depend on the binding of chaperones to specific sites within CFTR, which would be exposed in non-native states. A CFTR peptide library was used to identify binding sites for HSC70, DNAJA1 and DNAJA2, validated by competition and functional assays. Each chaperone had a distinct binding pattern, and sites were distributed between the surfaces of the CFTR cytosolic domains, and domain interfaces known to be important for channel assembly. The accessibility of sites to chaperones will depend on the degree of CFTR folding or unfolding. Different folded states may be recognized by unique combinations of HSC70, DNAJA1 and DNAJA2, leading to divergent biological effects.

## Introduction

Autosomal recessive cystic fibrosis (CF) is one of the most common lethal genetic diseases in the North American and European populations^1^. Typical outcomes include meconium ileus in newborns, pancreatic insufficiency, and recurrent lung infection due to bacterial colonization with uncontrolled inflammation, leading to the airway destruction and respiratory failure, the predominant cause of mortality in CF patients^2^. CF is caused by mutations in the *ABCC7* gene encoding the Cystic Fibrosis Transmembrane conductance Regulator (CFTR), a transmembrane channel that has a critical role in regulating transepithelial movement of water and electrolyte in epithelial cells. CFTR allows the flow of Cl^−^ and HCO_3_^−^ ions to maintain hydration, for example in lung airways. CF mutations in the channel render it dysfunctional or unstable. The most common mutation in CFTR is ΔF508, but many others are known^2,3^.

**Figure 1:**
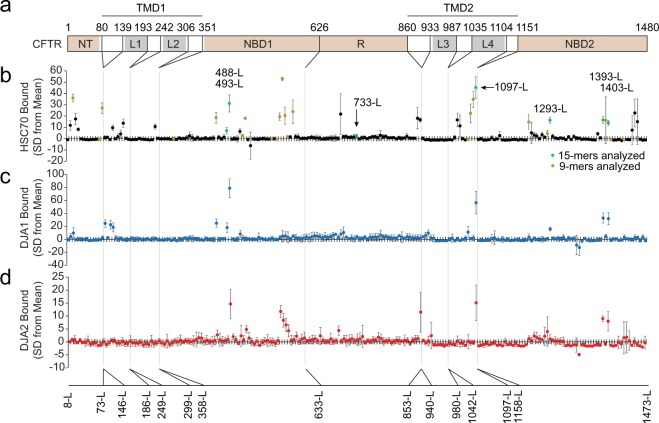
Chaperone binding to CFTR. (**a**) Diagram of CFTR domain structure. Residue numbers at the boundaries of the peptide library are marked. Cytosolic domains are in brown, loops are in grey, transmembrane helices and extracellular regions are in white. NT, N-terminal extension; L1 to L4, loop 1 to loop 4; NBD1 and 2, nucleotide binding domain 1 and 2; TMD1 and 2, transmembrane domain 1 and 2; R, regulatory region. Bottom, the corresponding peptide number in the CFTR sequence is marked. **(b**–**d)** A library of biotinylated 15-mer peptides with a 10-residue overlap between them, representing CFTR cytosolic sequences was immobilized on streptavidin plates. 2 μM purified His-tagged HSC70 **(b)** was bound in the presence of ATP, DJA1 **(c)** and DJA2 **(d)** in the absence of ATP, and bound chaperones quantified by ELISA detecting the His-tag, n ≥ 3. Binding to each peptide is shown as the number of standard deviations (SD) from mean binding values. Peptides analyzed in later experiments as 15-mers or 9-mers are marked in green and labeled, peptides represented by 9-mers and analyzed in later experiments are marked in gold. Error bars show standard deviations from the mean.

CFTR is a member of the ATP-binding cassette (ABC) transporter superfamily with the typical two transmembrane domains (TMD1 and TMD2), alternating with two cytosolic nucleotide-binding domains (NBD1 and NBD2) (Fig. 1a). Each TMD contains six transmembrane helices and two cytosolic loops (L1 and L2 in TMD1, L3 and L4 in TMD2). There is an additional N-terminal (NT) extension in the cytosol, and a unique regulatory (R) region lies between NBD1 and TMD2 (Fig. 1a)^3^. Homology models and structural studies including recent high-resolution cryo-EM structures of the full-length channel, established the arrangement of its domains^4–7^. The NT region packs onto the sides of L1 and L4, NBD1 is assembled onto the tips of L1 and L4, and NBD2 onto L2 and L3^4–7^. In the unphosphorylated nucleotide-free state, the channel is closed at the extracellular side, the NBDs are separate from each other, and R sits in the cleft between the NBDs^6^. In the phosphorylated ATP-bound state, NBD1 and NBD2 contact each other, the R region is displaced and unstructured, allowing the channel to open^7^.

The folding of CFTR begins during translation and translocation at the endoplasmic reticulum (ER), followed by a relatively slow post-translational folding and cooperative domain assembly^8–10^. Assembly of the NT onto the channel is critical for its regulation^11–13^. The CF mutation ΔF508 in NBD1 destabilizes the domain, and disrupts contacts with L4 and NBD2^8,14–16^. Folding of even wild-type CFTR is thought to be inefficient, and quality control ER-associated proteasomal degradation (ERAD) of misfolded CFTR comparatively fast, such that much of newly synthesized CFTR does not reach the plasma membrane (PM) in many cell types^17–19^. In addition, misfolded CFTR at the PM is directed to lysosomal degradation by the peripheral quality control machinery^20,21^.

Considering that ~70% of CFTR extends into the cytosol, its folding and degradation depend on cytosolic chaperones^22^. HSP90α and β are required for biosynthetic CFTR folding, and their regulatory co-chaperone AHA1 affects the extent of ERAD^23,24^. HSC70 (HSPA8), the constitutive form of HSP70 (HSPA1), promotes the folding of CFTR together with its J-domain co-chaperone DNAJA1 (DJA1)^25,26^. HSC70 and DJA1 bind to NBD1 in newly synthesized CFTR, and this association is lost during CFTR maturation^26^. However, HSC70 in complex with the E3 ubiquitin ligase CHIP selects misfolded CFTR both for ERAD, and for clearance from the PM^20,27,28^. HSC70 additionally promotes ERAD of CFTR dependent on the membrane-anchored E3 ligase RMA1^29^. How these opposite roles of HSC70 are balanced, remains unclear.

HSC70, like other highly conserved HSP70 family members including *Eschericia coli* DnaK, typically binds to short 7-residue peptide sequences with hydrophobic character and a preference for positive charges at one end^30–33^. It cycles between an ATP-bound state in which peptide substrate exchanges rapidly, and post-hydrolysis ADP-bound state which binds substrate tightly^34^. DJA1 and its close homolog DNAJA2 (DJA2) are both abundant J-domain co-chaperones, that bind to non-native polypeptide substrates and transfer them to HSC70, by stimulating ATP hydrolysis by HSC70^35^. Their well studied orthologs DnaJ in *E. coli* and Ydj1 in *Saccharomyces cerevisiae* bind short hydrophobic peptides like DnaK^36–39^, and evidence suggests DJA1 and DJA2 have a similar preference^40,41^.

DJA1 and DJA2 generally promote folding, but have distinct biological roles, and sometimes contradictory functions. DJA1 assists the folding of newly synthesized CFTR, as well as the hERG/Kv11.1 K^+^ channel and AID/AICDA cytidine deaminase in the cytosol^26,42,43^. At the same time, DJA1 promotes the lysosomal degradation of misfolded CFTR from the PM by the HSC70-CHIP mechanism^20^. In contrast, DJA2 cooperates with HSC70 to re-fold and stabilize partially unfolded CFTR mutants in phospholipid bilayers and at the PM, respectively^44^. This activity of DJA2 is comparable to re-folding of the model substrate luciferase, such that DJA1 is unable to substitute^45,46^. Recent findings suggest that DJA2 also promotes CHIP-mediated ERAD of CFTR^47^. How these conflicting outcomes are determined remains largely unknown.

It is possible that different folded states of CFTR expose different sequences which are recognized by HSC70, DJA1 and DJA2. We therefore hypothesize that each chaperone binds to distinct sets of sites within CFTR. Some chaperone binding patterns on non-native CFTR may be productive in assisting folding, while other unproductive patterns may increase probabilities of polyubiquitination by CHIP. To identify chaperone binding sites, a peptide library covering cytosolic CFTR regions was screened for binding by HSC70, DJA1 and DJA2, first by direct interaction, and then by competition assays. HSC70 sites were identified in the NT, NBD1, L4 and NBD2; DJA2 binding was similar but not identical, but DJA1 binding was unexpectedly more selective. Some chaperone sites would only be accessible during early folding stages, and also corresponded to parts of the structure important for stability. A different set of sites would be exposed in partially unfolded mature CFTR. Our results suggest how the same machinery of HSC70, DJA1 and DJA2 binding to a single polypeptide could lead to various functional proteostasis outcomes.

## Results

### Identification of chaperone sites

The cytosolic regions of CFTR were represented by a library of 15-mer peptides, with a 10-residue overlap between them, each with an N-terminal linker and biotin group. The peptides were numbered by their middle residue and suffixed L for long, as opposed to short S peptides used later (Supplemental Table S1). The peptides were immobilized on streptavidin plates, then 2 μM purified HSC70, DJA1 or DJA2 were bound and quantified by modified ELISA using Ni^2+^ detection of the His-tagged chaperones. Incubation of HSC70 was conducted in the presence of ATP to allow nucleotide cycling and dynamic steady-state peptide binding, but DJA1 and DJA2 were incubated in the absence of nucleotide. Binding sites for HSC70 were observed at a number of locations (Fig. 1b). DJA1 bound at fewer sites (Fig. 1c), while DJA2 binding was closer to that of HSC70 (Fig. 1d). The peptide 1097-L from L4 was selected for further study as being most consistently bound by all three chaperones, and peptide 733-L in the R region with low binding used as a negative control (Fig. 1b–d). HSC70 binding in ATP was somewhat lower than in ADP or the absence of nucleotide (Supplemental Fig. S1a), and we used the more conservative steady-state binding conditions with ATP. The raw values of the background ELISA signal in the absence of chaperones were low compared to chaperone binding (Supplemental Fig. S1c), suggesting that false positives from the detection method were unlikely.

HSC70 binding was compared to the prediction of HSP70-family binding sites by the LIMBO algorithm, which is based on the consensus of several studies on *E. coli* DnaK^31^. The high conservation between HSC70 and DnaK (76% similarity in the substrate binding domains) suggested binding sites may also be similar. However, while some binding sites had high LIMBO scores including 1097-L, other high scoring sites were not bound (Supplemental Fig. S1d). Hydrophobicity^48^ was also not a good predictor, as most HSC70 binding sites were hydrophobic, but many hydrophobic sequences remained unbound (Supplemental Fig. S1d). There were some matches between HSC70 binding and the TANGO algorithm for β-sheet aggregation propensity^49^, but not with the DisEmbl prediction of disordered regions^50^ (Supplemental Fig. S1e).

**Figure 2:**
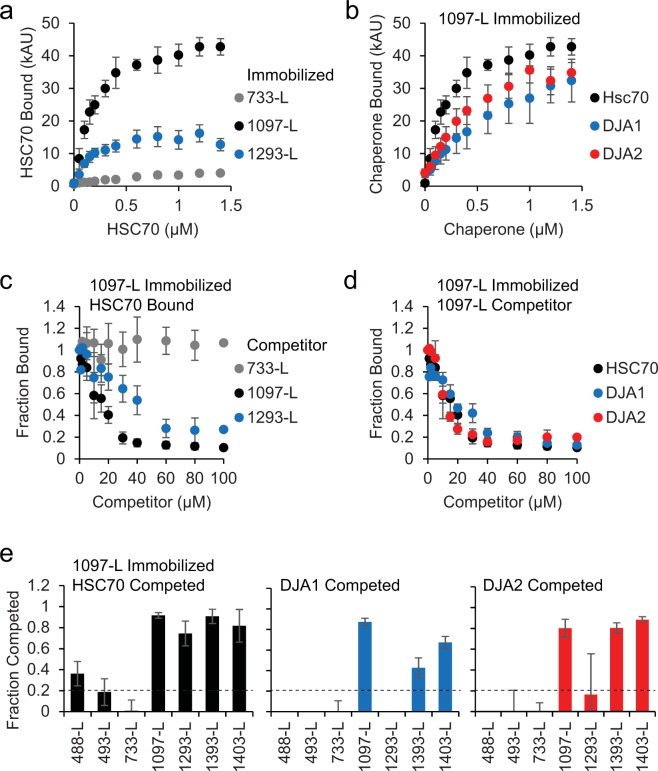
Binding and competition of chaperones. (**a**) The indicated peptide was immobilized, and increasing concentrations of HSC70 bound as in Fig. 1. HSC70 binding is represented as 1,000 arbitrary absorbance units (kAU), n ≥ 6. **(b)** Peptide 1097-L was immobilized and HSC70, DJA1 and DJA1 bound, n ≥ 6 for HSC70, n = 3 for DJA1 and DJA2. **(c)** Peptide 1097-L was immobilized, 1 μM HSC70 was incubated with increasing amounts of the indicated soluble competitor peptide, and binding quantified relative to that in the absence of competitor, n ≥ 12. **(d)** 1 μM HSC70, DJA1 and DJA2 were incubated with soluble competitor 1097-L and binding to immobilized 1097-L was quantified as in (**a**), n ≥ 8 for HSC70, n = 4 for DJA1 and DJA2. **(e)** 1 μM HSC70, DJA1 and DJA2 were incubated with 100 μM of the indicated competitor peptide and binding to immobilized 1097-L was quantified as in (**a**). The fraction competed was calculated as (1 – fraction bound), n ≥ 4. A screening cutoff (dashed line) was set as twice the standard deviation of the negative control 733-L. Error bars show standard deviations from the mean.

Specific binding is characterized by saturability, and competition. Therefore, binding to selected peptides was analyzed more closely, first by titrating the chaperones at increasing concentrations. HSC70 binding to immobilized 1097-L and 1293-L from NBD2 reached saturation at around 1 μM chaperone, and background binding to 733-L was low (Fig. 2a). Peptides 1393-L and 1403-L from NBD2 showed similar saturation behaviour (Supplemental Fig. S2a). The absolute amounts of DJA1 and DJA2 bound to 1097-L and the other peptides were comparable to each other and that of HSC70, also at or close to saturation above 1 μM (Fig. 2b, Supplemental Fig. S2b,c). The amounts of chaperones bound to 1097-L were higher than to the other peptides, possibly due to different accessibilities of the peptides on streptavidin, or a decrease in exposed peptides due to their aggregation during immobilization. Although the concentrations of immobilized peptide were not known, binding affinities could still be estimated from the saturation curves, as described below.

### Chaperone binding by competition

To further rule out false positive non-specific binding, and avoid problems of peptide accessibility on the plates, soluble peptides were used to compete chaperone binding to immobilized peptide. We incubated 1 μM HSC70 with up to 100 μM soluble peptide and assayed binding to immobilized 1097-L. Strong, essentially saturating competition was observed using soluble 1097-L and 1293-L, and not with negative control 733-L (Fig. 2c). Soluble 1097-L similarly competed the binding of DJA1 and DJA2 to immobilized 1097-L (Fig. 2d), consistent with the binding data (Fig. 2b). Soluble 1393-L and 1403-L also competed all of the chaperones well, while 1293-L only competed HSC70 (Fig. 2e, Supplemental Fig. S3a–c). Moreover, 488-L and 493-L competed poorly (Fig. 2e, Supplemental Fig. S3a) despite their binding by DJA1, HSC70 and DJA2, in the original screen (Fig. 1b–d), identifying them as false positives. These peptides may cause the chaperones to aggregate on the plate, giving the appearance of binding to immobilized peptide, but being unable to compete in solution. Thus, competition assays appeared to be more stringent than direct binding.

**Figure 3:**
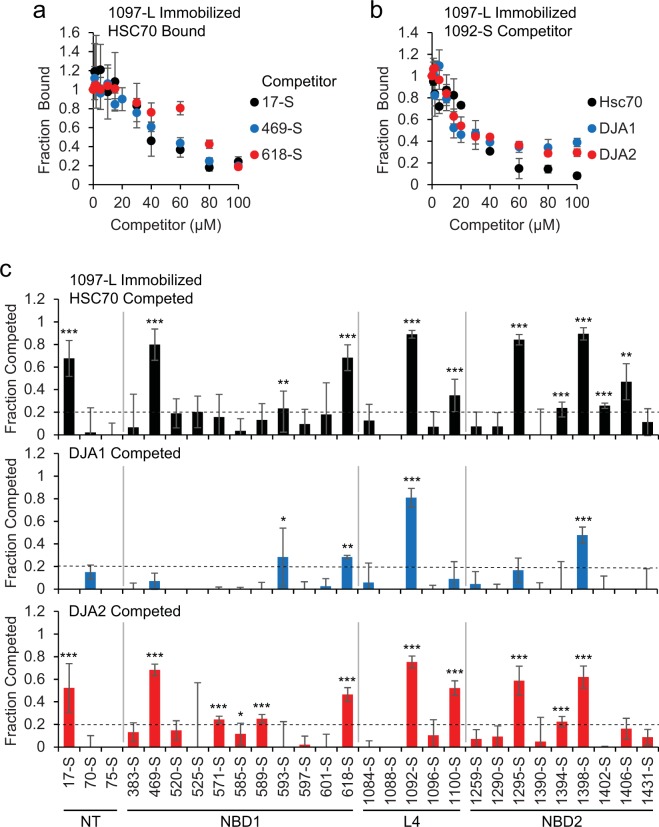
Competition identifies exact chaperone binding sites. (**a,b**) Peptide 1097-L was immobilized, and **(a)** HSC70 binding was competed with the indicated peptide as in Fig. 2c, or **(b)** HSC70, DJA1 and DJA2 binding was competed with peptide 1092-S as in Fig. 2d, n ≥ 3. **(c)** HSC70, DJA1 and DJA2 were bound to immobilized 1097-L and competed with the indicated peptide as in Fig. 2e, and fraction competed was calculated, n ≥ 4. A screening cutoff (dashed line) was set as twice the standard deviation of the negative control 733-L. The CFTR domain is marked below. Error bars show standard deviations from the mean, *p < 0.05, **p < 0.01, ***p < 0.001 compared to control without competitor.

The substrate binding pocket of HSC70 and homologs can fit a 7-residue peptide^30^, so we refined the long 15-mer peptides by breaking them into short (S) 9-mer peptides, again numbered by their central residue (Supplemental Table S2). Short peptides were chosen to represent strongly bound 15-mers from the peptide library results (Fig. 1b), and sequences with high LIMBO prediction scores, from the NT, NBD1 and NBD2 regions. We assayed the peptides for the ability to compete chaperone binding from immobilized 1097-L. The peptides 17-S from the CFTR NT, and 469-S and 618-S from NBD1, competed HSC70 well (Fig. 3a). The peptide 1092-S was a fragment of 1097-L, and it competed all three chaperones (Fig. 3b). In addition, HSC70 was competed by 1295-S and 1398-S; DJA1 was competed by 1398-S; and DJA2 was competed by 469-S, 1100-S and 1398-S (Supplemental Fig. S3d–f). A competition screen using a fixed concentration of all the S peptides was conducted, and identified distinct binding sites for each chaperone in the NT, NBD1, L4 and NBD2 of CFTR (Fig. 3c, summarized in Supplemental Table S2).

The binding affinities of peptides to HSC70, DJA1 and DJA2 were calculated from the binding and competition titrations, using methods established for displacement ELISAs^51^. The binding curves for 1097-L, 1393-L and 1403-L (Supplemental Fig. S2a–c) were transformed into linear functions (Supplemental Fig. S4a), and K_d_ calculated from the slopes. Affinities were between 0.1 μM for HSC70 binding 1097-L, and 11 μM for 1403-L (Table 1). The competition curves (Fig. 3, Supplemental Fig. S3) were then used to derive linear functions (Supplemental Fig. S4b–d) and K_d_ values. Most affinities were in the single micromolar range, between 1.25 and 5 μM for 1092-S and the three chaperones, up to 18 μM for 1398-S and DJA1 (Table 1, Supplemental Table S3). Because peptide immobilization on plates may interfere with binding of some chaperone molecules, the binding curves may underestimate affinity, while measurements with competing peptides in solution are likely to be more accurate. To validate these affinity calculations, the binding of fluorescently-labeled 1092-S by varying concentrations of HSC70, DJA1 and DJA2 was measured (Supplemental Fig. S5). Affinities were calculated from the half-maximal binding points, and were also in the single micromolar range (Table 1, Supplemental Table S3). These affinities are in agreement with previous reports of chaperone binding^36,52–57^.

**Table 1:** Binding affinities of chaperones for peptides (μM).

Peptide	HSC70	DJA1	DJA2
**From binding curves:**			
1097-L	0.058	0.167	0.075
1393-L	9.06	1.50	1.12
1403-L	11.1	1.49	1.19
**From competition curves:**			
1097-L	0.602	2.43	0.585
1293-L	2.02	—	—
1393-L	1.04	15.7	1.49
1403-L	2.66	9.15	0.994
17-S	1.76	—	—
469-S	1.56	—	4.82
618-S	3.73	—	—
1092-S	1.26	5.47	2.62
1100-S	—	—	8.33
1295-S	1.26	—	—
1398-S	0.900	18.3	4.45
**From fluorescence binding:**			
1092-S	6.17	3.97	8.46

### Functional validation of binding sites

**Figure 4:**
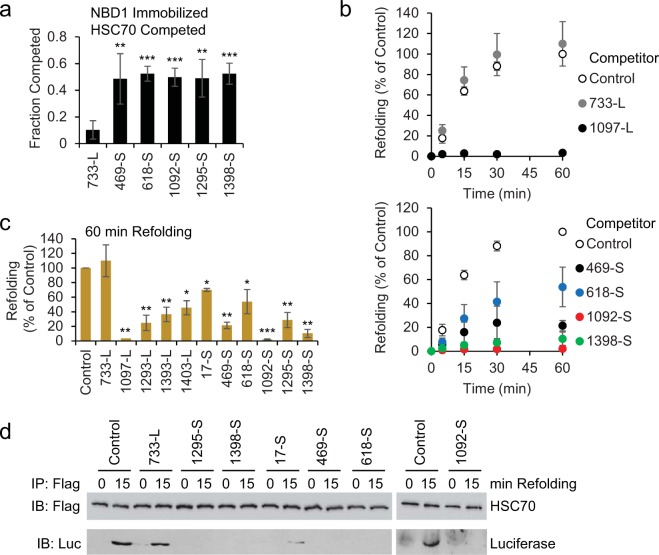
Substrate peptides disrupt HSC70 function. (**a**) Purified strep-tagged NBD1 was immobilized on streptavidin plates, and 1 μM HSC70 was bound in the presence of ATP as in Fig. 1a in the presence of the indicated competitor peptide, and quantified by ELISA. The binding was quantified relative to controls without peptide, and the fraction competed was calculated, n = 5, **p < 0.01, ***p < 0.001 compared to control without competitor. **(b)** Refolding reactions containing 4 μM each of HSC70 and DJA2, 100 nM guanidine denatured luciferase, and 100 μM of the indicated peptide, were monitored by luciferase activity over 60 min. Activities were quantified as a percentage of control reactions without peptide, n = 3. **(c)** Refolding after 60 min with the indicated peptide as in (**b**) was quantified, n = 3, *p < 0.05, **p < 0.01, ***p < 0.001 compared to control. **(d)** Refolding reactions as in (**b**) using Flag-tagged HSC70 were stopped at 0 or 15 min and immunoprecipitated with antibody against the Flag tag. HSC70 and bound luciferase were detected by Western blot.

We asked whether chaperone binding in the more native context of a full-length domain could still be competed by the peptides. CFTR NBD1 was purified with an Avi-tag^58^ and immobilized on streptavidin plates, incubated at 30 °C temperature to allow partial unfolding^15^, followed by HSC70 binding. In a direct comparison experiment, HSC70 binding to NBD1 was 73 ± 3% of that observed for 1097-L (n = 3, p < 0.01). When binding to NBD1 was conducted in the presence of soluble peptides, sequences from NBD1 (469-S and 618-S) competed the majority of HSC70, while the control 733-L did not (Fig. 4a). Peptides derived from L4 and ND2 (1092-S, 1295-S and 1398-S) were also effective competitors (Fig. 4a).

Next, to determine if the peptides were bound by the true substrate binding site of HSC70, we asked if they disrupted its chaperone function. The refolding of denatured luciferase requires functional substrate binding and ATPase activity of HSC70, as well as functional DJA2, *in vitro* and in cells^45,46^. As established, refolding by HSC70 and DJA2 reached completion by 60 min^46^, and a 25-fold excess of 1097-L blocked refolding entirely, while 733-L had no effect (Fig. 4b). Peptides 469-S, 1092-S and 1398-S also inhibited refolding (Fig. 4b). The inhibition observed with peptides 17-S, 618-S and 1295-S was partial but still significant (Fig. 4c).

To directly address whether peptides displaced the luciferase substrate from HSC70, the chaperone was immunoprecipitated at 0 and 15 min of the refolding reaction, and bound luciferase detected. In the untreated control and with 733-L, luciferase was substantially bound, but not in the presence of the inhibitory peptides (Fig. 4d). Therefore, the peptides are most likely bound in the HSC70 substrate binding site.

## Discussion

**Figure 5:**
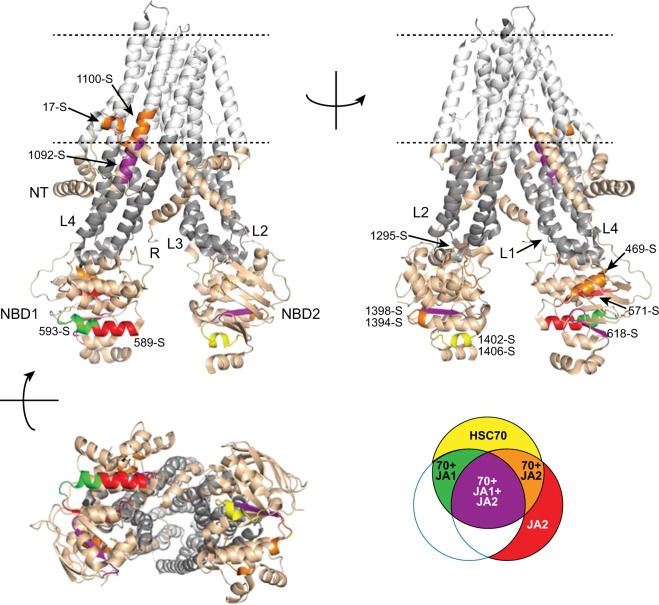
Location of chaperone binding sites on CFTR. The structure of nucleotide-free CFTR (PDB 5UAK) is depicted using PyMOL (v1.74 Schrödinger), domains labeled and coloured as in Fig. 1. The position of the membrane bilayer determined from the cryo-EM structure^59^ is shown by dashed lines. Chaperone binding sites from Fig. 3c are coloured according to the legend on the lower right. The peptide numbers corresponding to the chaperone sites are marked.

The chaperone binding sites (Fig. 3c) can be mapped onto the structure of CFTR (Fig. 5). With a couple exceptions, DJA2 bound to the same sites on CFTR as HSC70, but DJA1 was more selective. The sites cluster in a few locations: where NT assembles onto L4; at the interface between NBD1 and L4; the interface between NBD2 and L2; and within NBD1 and NBD2 (Fig. 5). However, some sites are relatively exposed in the native structure, while others are buried. The exposed sites are likely to be accessible for chaperone binding when WT CFTR is close to its native state – at later stages of biosynthetic maturation, representing the post-translational folding phase^8^, which is also characterized by the delayed dissociation of chaperones^26^. In addition, these sites may be also accessible during transient unfolding of the mature protein. Sites buried within the protein will only be accessible when the protein is mostly unfolded, at early stages of biosynthetic folding. Distinct sets of binding sites will be accessible at different stages of folding of the channel. Patterns of chaperone binding may therefore determine the specific fate of CFTR molecules.

The most exposed sites are in the NBDs. At the surface of NBD1 is the DJA2 site at peptide 589-S, and a DJA1 site at 593-S; at the surface of NBD2 is the HSC70 site at 1402-S to 1406-S. A potentially exposed region is the NT-L4 interface, with binding sites for HSC70 and DJA2 on both side at 17-S and 1100-S, and the L4 side is one of the few sites also bound by DJA1, at 1092-S (Fig. 5). A new analysis of transmembrane structures based on cryo-EM structures in detergent micelles^59^ indicates that 1100-S may be within a transmembrane helix and unaccessible to chaperones, but 1092-S will be exposed to the cytosol. Part of NT that includes 17-S may dip into the membrane, although not deeply (Fig. 5). It is possible that this region is bound by the chaperones during the insertion into the membrane and folding of the NT, which must be a later stage in folding because it requires prior assembly of L4 and L1. The chaperone sites may also become exposed in the cytosol by partial unfolding of mature CFTR. Interestingly, the NT region was found to interact with DNAJC5/CSP, a co-chaperone which has a substrate-binding site structurally unrelated to DJA1 or DJA2, but which also promotes ERAD of CFTR^60,61^. In general, these binding sites would be accessible to chaperones in near-native states of CFTR, after mild stress or even with regular thermal motion breathing of the CFTR structure.

Two sites are at interdomain contacts. The HSC70-DJA2 sites at 469-S and 1295-S are at the interfaces of NBD1-L4, and NBD2-L2, respectively (Fig. 5). Although they are partly buried in the native structure, separation of the NBDs from the loops during partial unfolding would make the sites available for binding. However, the NBDs could still remain largely folded while the sites are exposed. These relatively exposed DJA2 sites may be important for its unique function in refolding mature CFTR.

Other sites are buried inside the mostly hydrophobic cores of the NBDs. These are sites 571-S and 618-S in NBD1, and 1394-S to 1398-S in NBD2 (Fig. 5). Although 571-S is bound only by DJA2, the other sites are bound by HSC70, DJA2 and DJA1. The chaperones will likely recognize these sites when CFTR is first translated, to protect the hydrophobic sequences from aggregation. The sites would only be exposed in mature CFTR upon relatively severe stress. Prolonged exposure of the sites would signal gross misfolding, perhaps leading to polyubiquitination by CHIP and either ERAD or turnover from the PM.

The distribution of chaperone binding is consistent with previous work on the CFTR folding process. The assembly of the NT region is important for CFTR function, and the NT helps maintain the rigidity of the channel for its opening and closing^5^. The hydrophobic chaperone binding sequence in the NT is next to a charged region important for its assembly, as well as CFTR stability at the cell surface^11–13^. Also, the ΔF508 mutation disrupts the NBD1 interface with L4 and NBD2^8,14,15^. The same part of NBD1 is supported by the sequences at 571-S and 585-S to 593-S (Fig. 5), suggesting the chaperones may stabilize this labile part of the domain, especially at early stages in folding when NBD1 is largely in an extended conformation with these hydrophobic sequences exposed. Furthermore, the sites at 469-S and 571-S at the NBD1-L4 interface may become abnormally exposed upon misfolding caused by the ΔF508 mutation. Site 618-S, which is normally entirely buried inside NBD1, may also become partially exposed. Increased binding of chaperones to these sites could lead to different effects on the mutant compared to WT.

The distribution of HSC70 binding sites was sparser than expected, from both the LIMBO prediction, and overall hydrophobicity. In agreement, a recent study of HSC70 binding to Tau also found relatively few sites^41^. The inaccuracy of the LIMBO algorithm may be due to subtle differences in the substrate binding domain between HSC70 and *E. coli* DnaK^32,57,62,63^, or the inclusion of false positives in its data set. Our study identified too few sequences to be statistically useful in developing an HSC70-specific pattern. It appears that the actual sequence selectivity of HSC70 remains to be finally determined.

A recent study addressed the binding of peptides by the endoplasmic reticulum HSP70 paralog BiP, and its co-chaperone ERdj3 which is structurally related to DJA1 and DJA2, as well as ERdj4 and ERdj5 which are not structurally related outside of the J domain^64^. Interestingly, most of the peptides bound by ERdj3 were also bound by BiP^64^, and this parallels the similarity in binding we observe between HSC70 and DJA2. Because DJA1 binding is more selective, a strong similarity in sequence preference between HSC70 and its co-chaperones is not strictly required for chaperone function. Indeed, peptide binding of ERdj4 and ERdj5 was divergent from BiP – they recognized fewer peptides and had a preference for sequences predicted to be aggregation prone^64^. However, ERdj4 and ERdj5 have particular roles in degradation, while DJA1 generally supports folding, like DJA2 and ERdj3. One possibility is that the different preferences of DJA1 and DJA2 allow the HSC70 chaperone system to access a wider range of substrate polypeptides, in different states of folding.

HSP70-family chaperones are thought to act on polypeptides with an unfolding mechanism^65^. Single-molecule experiments found that DnaK binding to multiple sites on a polypeptide is sufficient to cause its unfolding, but it involved up to seven molecules of DnaK bound to 35 kDa rhodanese^66^. Our results suggest that HSC70 binding to CFTR is not as dense, for example with three binding sites in the 30 kDa NBD1 (Fig. 5). Another single-molecule study provided evidence that DnaK and DnaJ cooperatively unfold polypeptides^67^, and this model may be more consistent with our findings. Speculatively, different DJA1 and DJA2 binding sites could lead to different pathways or efficiencies of unfolding by HSC70.

There is growing evidence that DJA1 and DJA2 are functionally distinct. DJA1 promotes the biosynthetic folding of CFTR, hERG, AID and cytosolic-oriented CD38^26,29,42,43,68^. DJA2 assists luciferase, trimeric G proteins, Tau maintenance, and RISC assembly^41,46,69,70^, but at the same time, the degradation of hERG and CD38^43,45,68^. To explain this distinction, we previously found differences in their transfer of substrate polypeptide to HSC70^45^. Now, we also propose that binding of DJA1 and DJA2 to different sites within the same substrate, can result in divergent functional outcomes. Furthermore, the DJAs form a transient ternary complex with substrate and HSC70 during J-domain stimulation of ATP hydrolysis by HSC70^34^. Some sets of chaperone binding to conformations of a polypeptide may be productive for its folding of a polypeptide, while others are not. Dependence of a protein on DJA1 or DJA2 would be determined not just by binding, but by where in the sequence they bind, along with the coordinated transfer to HSC70. Future experiments will address the binding of HSC70, DJA1 and DJA2 to other polypeptides, and how the patterns relate to folding and unfolding rates, and to quality control degradation in cells.

## Methods

### Proteins and peptides

HSC70, DJA1 and DJA2 were purified as His-tagged proteins, as described^46^. HSC70 was also amplified by PCR with an N-terminal FLAG tag, inserted into pPROEXHTa (Invitrogen) and purified as described. HSC70 was stored in 100 mM KOAc, 20 mM HEPES-KOH pH 7.5, 5 mM MgOAC_2_ (buffer G), DJA1 and DJA2 in 500 mM NaCl, 20 mM HEPES-KOH pH 7.5, 5 mM MgOAC_2_ (buffer H). Avi-tagged CFTR NBD1 was purified as described^58^.

The CFTR peptide library was designed as 15-mers with 10-residue overlaps covering the cytosolic sequences as in Fig. 1a and Supplemental Table S1. Synthetic peptides (Mimotopes, PepSet purity) contained an N-terminal biotin followed by linker sequence GSGS and the 15-mer sequence. Soluble peptides (Lifetein) were synthesized unmodified at >85% purity, sequences in Supplemental Table S1 and S2. Fluorescent peptide 1092-S was fused to the linker sequence SG and an N-terminal 6-carboxyfluorescein (FAM) label. Peptides were reconstituted in DMSO or water.

### ELISA

For peptide binding, streptavidin-coated black 96-well plates (Nunc Maxisorp, ThermoFisher) were pre-rinsed with PBS containing 0.05% Tween-20 (PBST), and 5 µM biotinylated peptides in 100 µl PBS were bound overnnight at 4 °C with agitation. Plates were washed with PBST and blocked with PBST containing 0.5% bovine serum albumin (BSA) for 1 h. After removal of blocking solution, 100 µl reactions containing chaperones at indicated concentrations were bound for 45 min at room temperature with agitation. HSC70 reactions were in buffer G containing 1 mM ATP, DJA1 and DJA2 reactions were in buffer H. For competition assays, chaperones and peptides were mixed at indicated concentrations immediately before the reactions were added to the plates. For detections, the plates were washed in PBST and 5 μM His-Probe-HRP conjugate (ThermoFisher) in 100 μL PBST added for 15 min with agitation. After washing in PBST, plates were developed using Amplex Red (ThermoFisher) according to manufacturer’s instructions, in a fluorescence plate reader (Tecan M1000).

For NBD1 binding, 100 nM of strep-tagged NBD1 in 100 µl of 150 mM NaCl, 50 mM Na_2_HPO_4_ pH 7.5, 1 mM ATP, and 40 µM DTT (buffer N), was bound to streptavidin plates for 1 hour at 4 °C with agitation. Plates were washed with PBST and incubated in buffer N at 30 °C in a water bath for 5 min. Then, plates were blocked in PBST containing 0.5% BSA, followed by the addition of HSC70 binding reactions, and detection as above.

### Sequence analysis

Hydrophobicity analysis using the Abraham & Leo scale^48^ was conducted with ExPasy ProtScale, using a 15-residue window to match the peptide library, without normalization and with the linear weight variation model. The LIMBO DnaK binding analysis^31^ used the best overall prediction mode. The TANGO prediction of aggregation propensity^49^ and the DisEmbl prediction of unstructured regions^50^ was for pH 7.5, temperature 298.15 °K, ionic strength 0.2 M, concentration 0.0001 M, with other parameters set as default.

### Luciferase refolding

Refolding of guanidine denatured luciferase with HSC70 and DJA2 was as described^46^. 100 µl reactions contained 4 μM each of HSC70 and DJA2, 2 mM ATP and 100 μM luciferase in buffer G, with the indicated concentrations of peptides. To monitor refolding, luciferase activity was measured in a luminometer (Berthold Sirius) with the luciferase assay kit (Promega). For co-IPs, 20 μL reaction aliquots were stopped by dilution at 4 °C to 500 μL with buffer containing 0.1% Triton X-100, 0.2% BSA and 0.04 mg/mL apyrase (Sigma). Reactions were incubated with FLAG-M2 magnetic beads (Sigma) for 1 h at 4 °C with agitation, then beads were isolated and washed with buffer G containing 0.1% Triton X-100, and eluted with 40 μL of 1 μg/mL FLAG peptide in the same buffer. Samples were analyzed on Western blots detected with antibodies against luciferase (Sigma, L2164) and the FLAG-M2 tag (Sigma, F1804).

### Affinity calculations

Affinities were determined separately from binding curves and from competition curves^51^. Data from binding of chaperones to immobilized peptides (Fig. 2a,b and Supplemental Fig. S2a–c) were fit to the equation 1/(1 − i) = (C/i)/K_d_^B^ − b, where i is the relative saturation, C is the chaperone concentration (1 μM), K_d_^B^ is the apparent dissociation constant from the binding data. In the plot of 1/(1 − i) against (C/i), the slope is 1/K_d_^B^, and −b is the intercept. The maximum amount of chaperone bound to 1097-L was set as i = 1. Data from competition of chaperones with peptides from immobilized 1097-L (Figs. 2c,d and 3a–b, Supplemental Fig. S3) were fit to the equation bi + (C/K_d_^B^)/i = (1/K_d_^C^)D + e, where −b is the intercept from the 1097-L binding curves, i is the relative saturation, C is the chaperone concentration (1 μM), K_d_^B^ is the dissociation constant from 1097-L binding curves, K_d_^C^ is the dissociation constant of the competing peptide, and D is the concentration of competing peptides. In the plot of bi + (C/K_d_^B^)/i over D, the slope is 1/K_d_^C^, and e is the intercept. The amount of chaperone bound in the absence of competitor was set as i = 1. The curves were fit by linear regression in Excel, and confirmed by the R^2^ correlation coefficients (Supplemental Table S3).

### Fluorescent peptide binding

0.5 μM FAM-1092-S was incubated with chaperones at the indicated concentrations for 45 min. HSC70 was incubated in buffer G, DJA1 and DJA2 were incubated in buffer H. The fluorescence signal for each condition was measured at 492 nm wavelength excitation, 520 nm wavelength emission, and 5 nm slit width, using a Horiba FluoroMax spectrofluorometer. Data were collected with the FluorEssence software (Horiba). Dissociation constants (K_d_) were determined by nonlinear regression fit of the averaged data to a one site – total binding model, using GraphPad Prism 8. The 95% confidence intervals and R^2^ correlation coefficients are reported (Supplemental Table S3).

### Statistics

Established statistical methods^71–73^ were used to analyze the data for chaperone binding to the peptide library. First, biological and statistical outliers were identified with the Tukey method in two rounds, for biological and then statistical outliers. The remaining values were confirmed to be normally distributed using Shapiro-Francia tests, Q-Q plots and Kolmogorov-Smirnov tests, and the means and standard deviations calculated. The screening cut-off was evaluated for a one-sided 90% lower confidence limit for the 95^th^ percentile and based on values of t-distribution was determined to be 1.645 standard deviations. Binding data were represented as multiples of the standard deviations from the normal means. Binding in the presence of competitor peptides were shown as a fraction of binding in the absence of competitor, or the fraction competed calculated as 1 – fraction bound. Values were compared using a two-tailed unpaired Student’s t-test assuming unequal variance.

## Data Availability

All data generated or analysed during this study are included in this article and its Supplementary Information files, or are available from the corresponding author upon request.

## Supporting Information

Supporting Information.
Supporting Information 2.
