# Supplementary material for: Selective Binding of HSC70 and its Co-Chaperones to Structural Hotspots on CFTR
Source: Sci Rep. 2020 Mar 6;10:4176. doi: 10.1038/s41598-020-61107-x (PMC7060200; doi:10.1038/s41598-020-61107-x)

Selective Binding of HSC70 and its Co-Chaperones to Structural Hotspots on CFTR

Imad Baaklini[1], Conrado de Campos Gonçalves[1], Gergely L. Lukacs[1,2], and Jason C. Young[1,*]

[1]McGill University, Department of Biochemistry, Montreal, H3G 1Y6, Canada

[2]McGill University, Department of Physiology, Montreal, H3G 1Y6, Canada

*jason.young2@mcgill.ca

Supplemental Figure Legends

Figure S1. HSC70 binding sites on CFTR and polypeptide properties. (a) Peptide 1097-L was immobilized and 1 μM HSC70 bound in the absence of nucleotide, or in the presence of ADP or ATP as in Fig. 1b. Binding is quantified as a fraction of the maximum binding observed, n≥4. **(b)** Domain structure of CFTR as in Fig. 1. Bottom, the corresponding peptide number in the CFTR sequence is marked. **(c)** The raw HSC70 binding signals from Fig. 1b are plotted together with the negative control signals detected in the absence of His-tagged chaperones, represented as 1,000 arbitrary absorbance units (kAU), n≥4. **(d)** Hydrophobicity of CFTR using a 15-residue window is plotted together with predictions of HSC70 binding by LIMBO. **(e)** Prediction of disordered sequences by DisEmbl is plotted together with predictions of β-sheet aggregation propensity by TANGO. Error bars show standard deviations from the mean.

Figure S2. Titration of chaperone binding to peptides. (a to c) The indicated peptide was immobilized, and increasing amounts of HSC70 **(a)**, DJA1 **(b)** and DJA2 **(c)** were bound as in Fig. 1. Binding is represented as 1,000 arbitrary absorbance units (kAU), n≥6 for HSC70, n=3 for DJA1 and DJA2. Error bars show standard deviations from the mean.

Figure S3. Competition of chaperone binding. (a to c) Peptide 1097-L was immobilized, 1 μM HSC70 **(a, d)**, DJA1 **(b, e)** and DJA2 **(c, f)** binding in the presence of the indicated competitor peptide was quantified as in Fig. 2c and d. Long 15-mer **(a to c)** and short 9- or 10-mer peptides **(d to f)** were used, n≥3. Error bars show standard deviations from the mean.

Figure S4. Binding and competition curves for affinity calculations. (a) Chaperone binding data from Supplemental Fig. S2a to c, were plotted as 1/(1-i) over C/i, where i is the fraction saturated, C is the concentration of chaperone, and binding affinity K_d(B) is 1/slope. **(b to d)** Chaperone competition data from Fig. 3 and Supplemental Fig. S3 for HSC70 **(b)**, DJA1 **(c)** and DJA2 **(d)**, n≥3, were plotted as $b_i+(C/K_d(B))/i$ over D, where -bi is the the intercept for 1097-L binding in Supplemental Fig. S4a, C is the concentration of chaperone, Kd(B) is binding affinity for 1097-L, i is the fraction saturated, D is concentration of competitor peptide, and competition affinity Kd(C) is 1/slope. See Methods for details, and Supplemental Table S2 for slopes and R^2 correlation coefficients.

Figure S5. Chaperone binding to fluorescently-labeled peptide. (a to c) 0.5 μM FAM-labeled 1092-S was incubated with the indicated concentrations of HSC70 **(a)**, DJA1 **(b)** and DJA2 **(c)**. The fluorescence signal at 492 nm excitation and 520 nm emission was measured in abritary units (AU), n≥8. Curves fit by linear regression to a single-site binding model are shown. Error bars show standard deviations from the mean.

Table S1. L Peptides. The names, start and end residues and CFTR domain of the 15-mer L peptides are listed.

Table S2. S Peptides. The names, sequences, middle residues and lengths of the 9- and 10-mer S peptides are listed. The names are coloured by chaperone binding according to the legend, the same as in Fig. 5.

Table S3. Affinity Calculations. The dissociation constants of peptides binding to HSC70, DNAJA1 and DNAJA2 were calculated from the ELISA data in Supplemental Fig. S4a and Supplemental Fig. S4b-d, and the fluorescence data in Supplemental Fig. S5. The R^2 correlation coefficients and other parameters from the ELISA calculations are listed. The R^2 correlation coefficients and 95% confidence interval (CI) from the fluorescence calculations are listed.

Figure S1

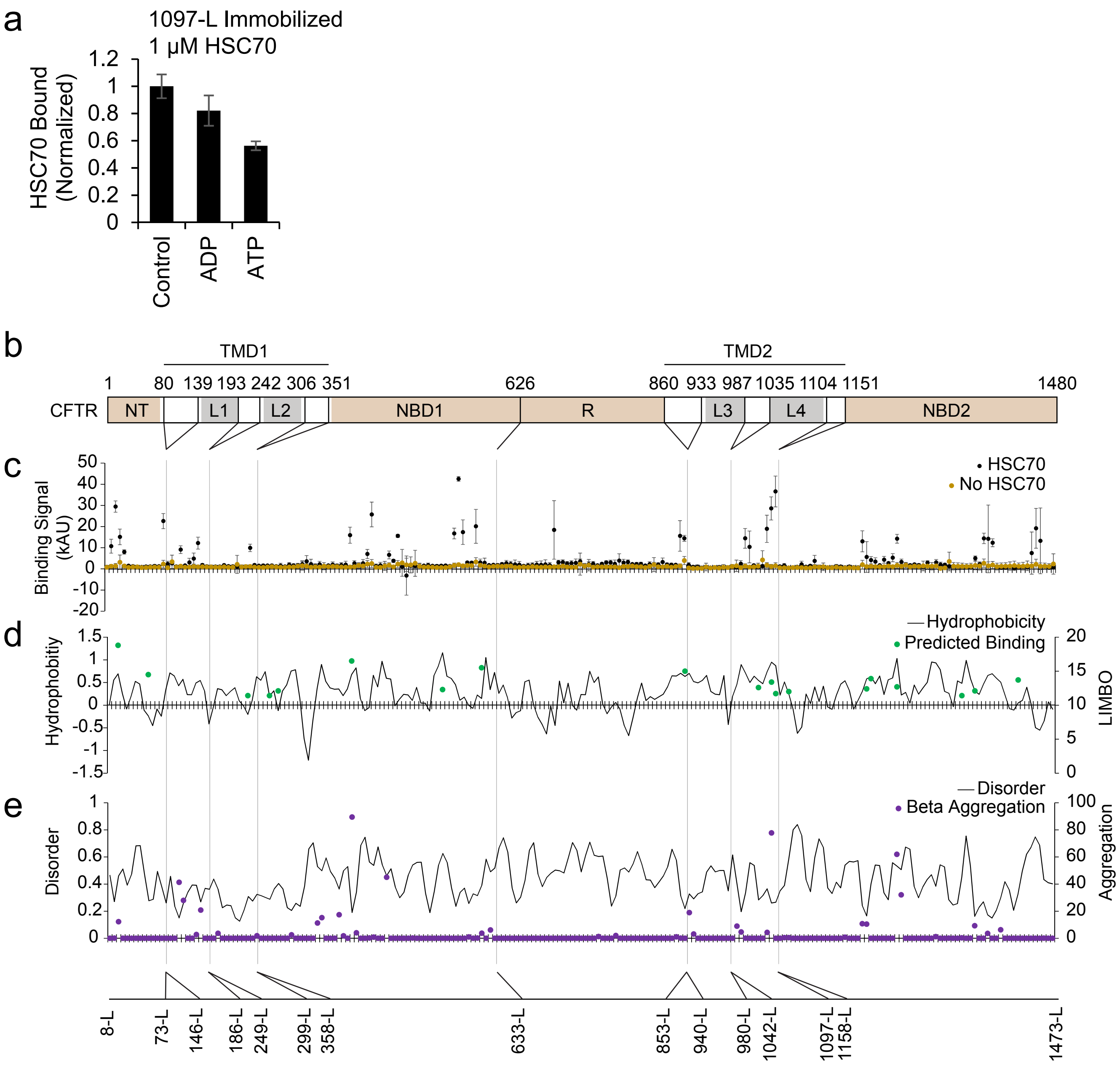

a
1097-L Immobilized
1 µM HSC70
HSC70 Bound (Normalized)
Control
ADP
ATP
b
TMD1
TMD2
CFTR
NT
L1
L2
NBD1
R
L3
L4
NBD2
1 80 139 193 242 306 351 626 860 933 987 1035 1104 1151 1480
c
Binding Signal (kAU)
HSC70
No HSC70
d
Hydrophobicity
Predicted Binding
LIMBO
e
Disorder
Beta Aggregation
Aggregation
8-L
73-L
146-L
186-L
249-L
299-L
358-L
633-L
853-L
940-L
980-L
1042-L
1097-L
1158-L
1473-L

Figure S2

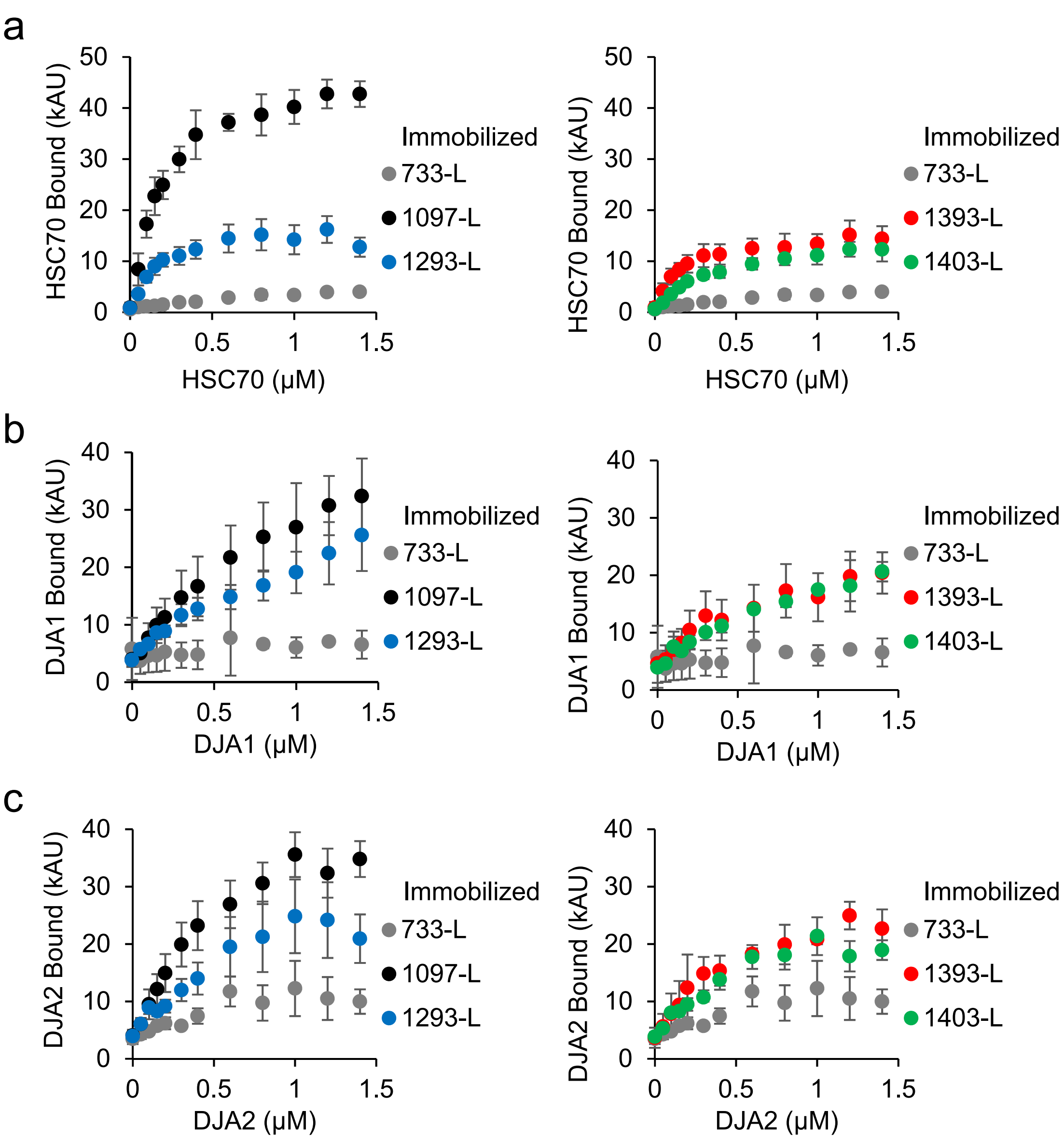
a
HSC70 Bound (kAU)
HSC70 (µM)
Immobilized
733-L
1097-L
1293-L
1393-L
1403-L
b
DJA1 Bound (kAU)
DJA1 (µM)
c
DJA2 Bound (kAU)
DJA2 (µM)

Figure S3

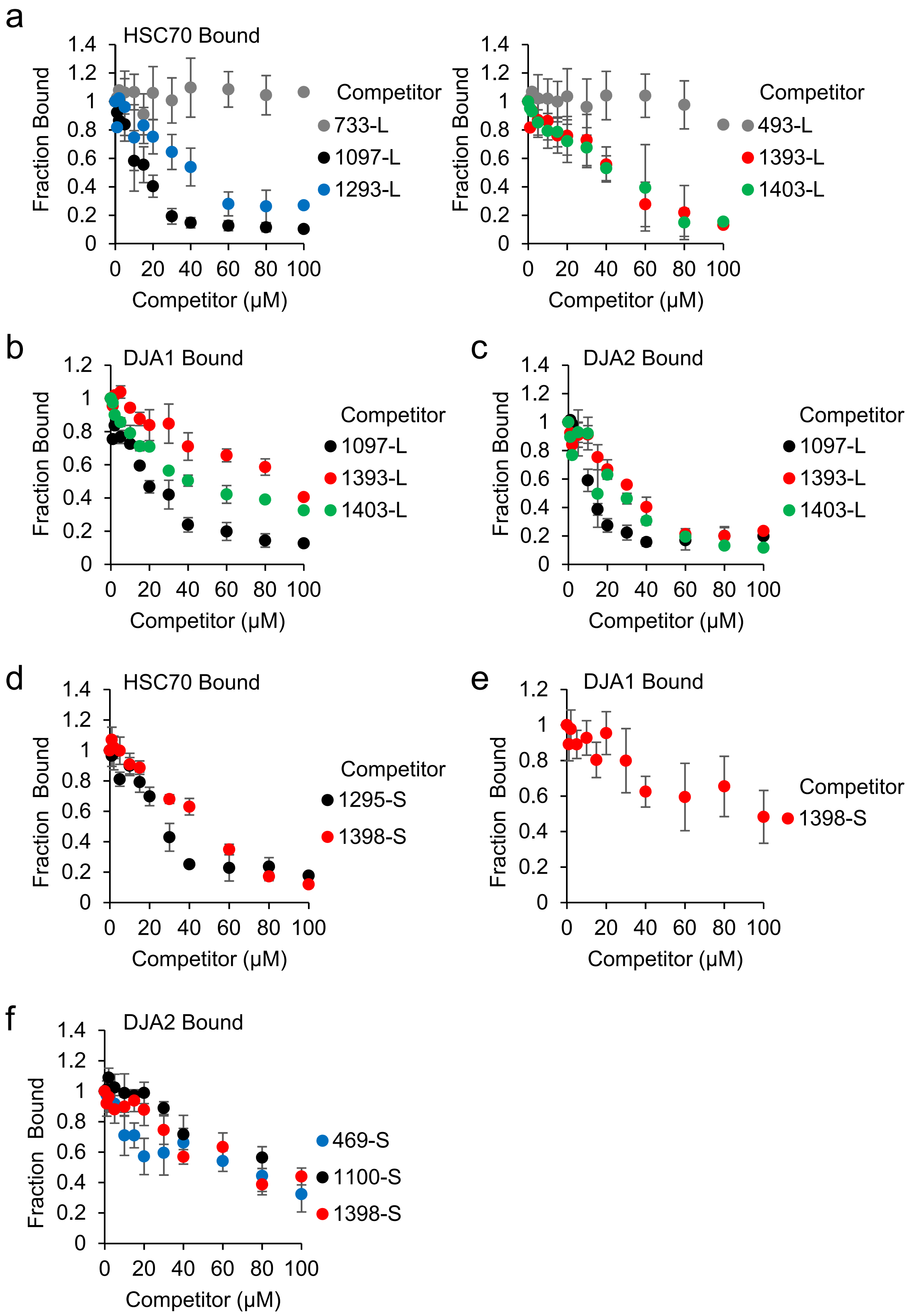
a
HSC70 Bound
Fraction Bound
Competitor (μM)
Competitor
733-L
1097-L
1293-L
493-L
1393-L
1403-L
b
DJA1 Bound
1097-L
1393-L
1403-L
c
DJA2 Bound
d
HSC70 Bound
1295-S
1398-S
e
DJA1 Bound
f
DJA2 Bound
469-S
1100-S
1398-S

Figure S4

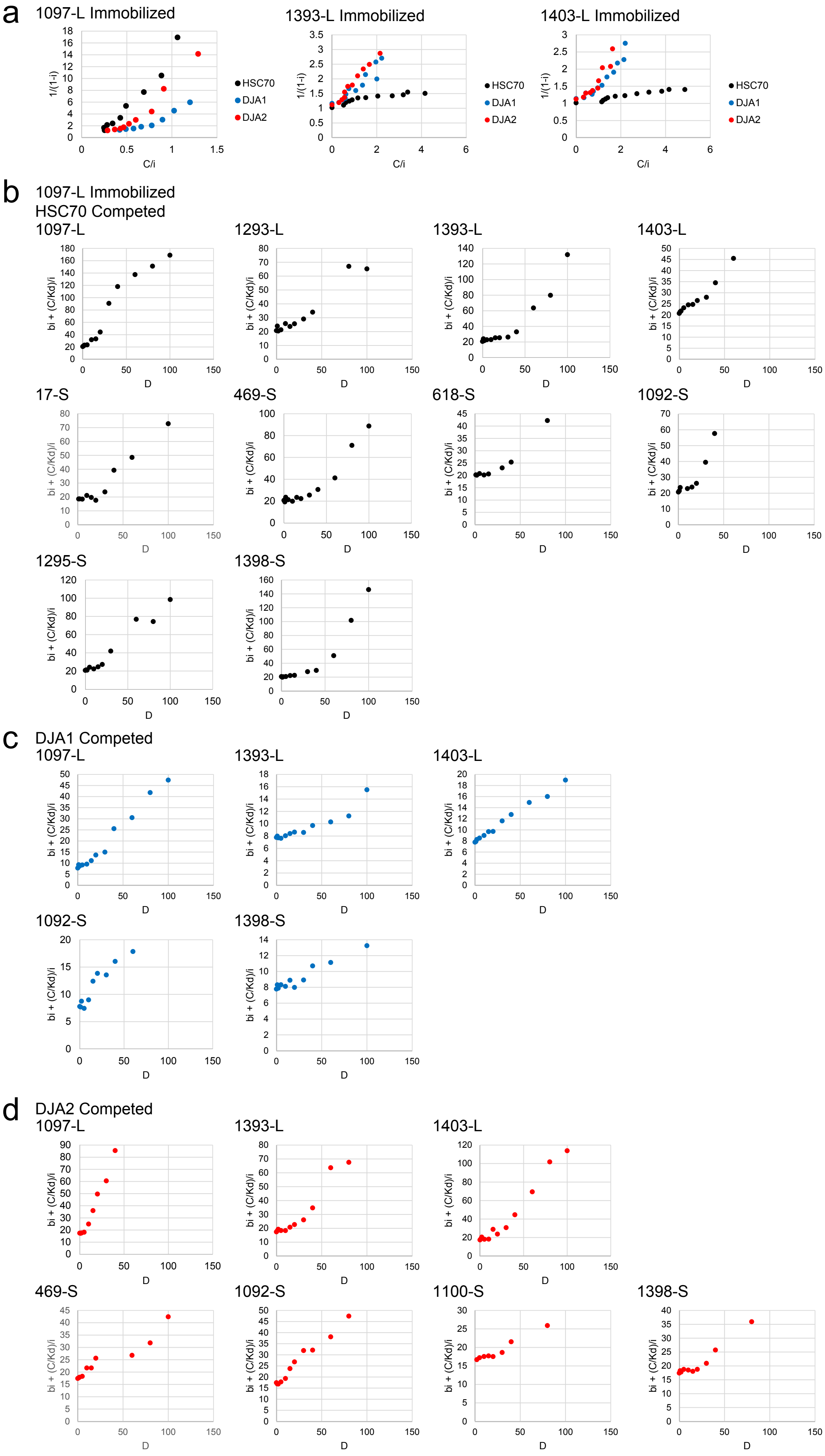
a
1097-L Immobilized
1393-L Immobilized
1403-L Immobilized
1/(1-i)
C/i
HSC70
DJA1
DJA2
b
1097-L Immobilized
HSC70 Competed
1097-L
1293-L
1393-L
1403-L
17-S
469-S
618-S
1092-S
1295-S
1398-S
bi + (C/Kd)/i
D
c
DJA1 Competed
1097-L
1393-L
1403-L
1092-S
1398-S
d
DJA2 Competed
1097-L
1393-L
1403-L
469-S
1092-S
1100-S
1398-S

Figure S5

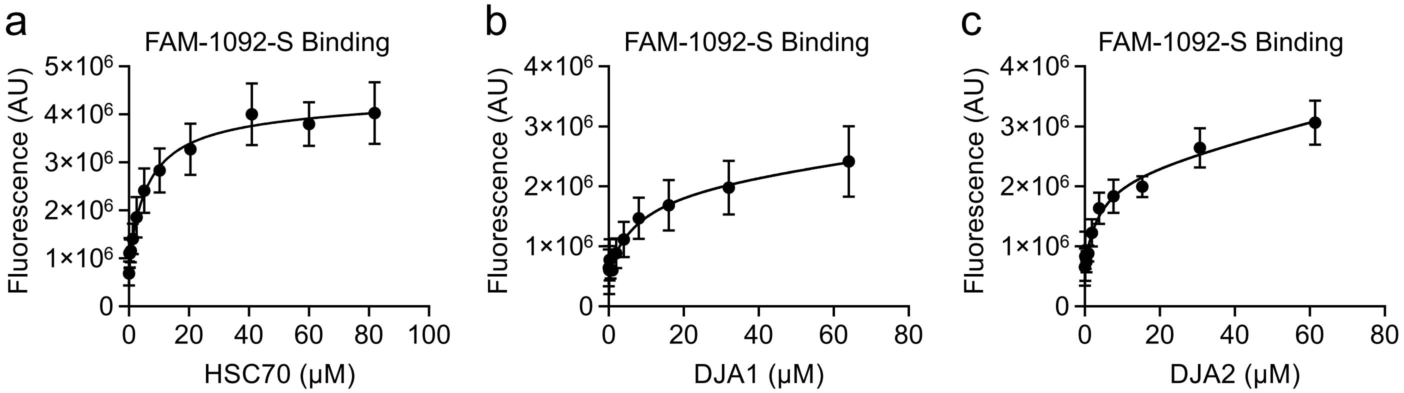

a
FAM-1092-S Binding
Fluorescence (AU)
HSC70 (µM)
b
FAM-1092-S Binding
Fluorescence (AU)
DJA1 (µM)
c
FAM-1092-S Binding
Fluorescence (AU)
DJA2 (µM)

Supplementary File:
Original Western blots for Fig. 4d

IP: Flag
IB: Flag

IB: Luc

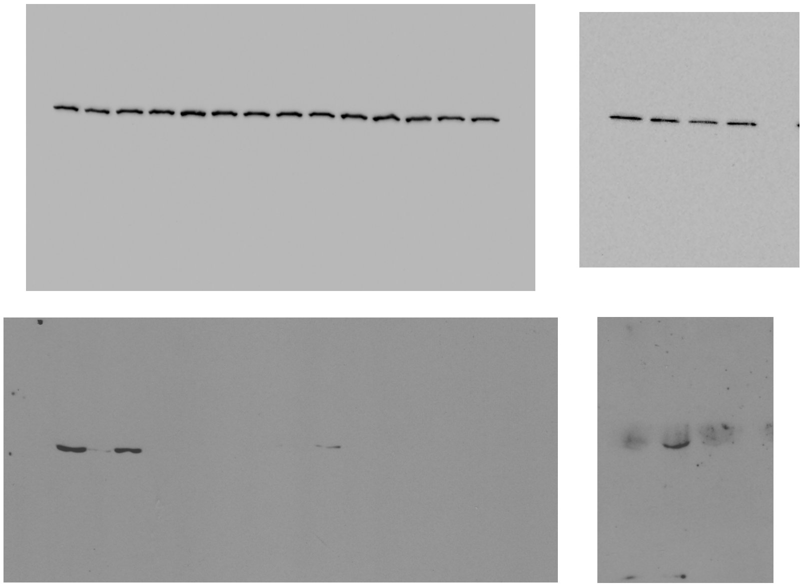

Supplement: Supplementary file 2 — Supporting Information 2. [file 41598_2020_61107_MOESM2_ESM.pdf]
